# Parents’ Experiences with Home-Based Oral Chemotherapy Prescribed to a Child Diagnosed with Acute Lymphoblastic Leukemia: A Qualitative Study

**DOI:** 10.3390/curroncol28060372

**Published:** 2021-11-01

**Authors:** Étienne Camiré-Bernier, Erwan Nidelet, Amel Baghdadli, Gabriel Demers, Marie-Christine Boulanger, Marie-Claude Brisson, Bruno Michon, Sophie Lauzier, Isabelle Laverdière

**Affiliations:** 1 Faculty of Pharmacy, Université Laval, Québec, QC G1V 0A6, Canada; etienne.camire-bernier@chudequebec.ca (É.C.-B.); erwan.nidelet@chudequebec.ca (E.N.); 2 Department of Pharmacy, CHU de Québec-Université Laval, Québec, QC G1V 4G2, Canada; marie-christine.boulanger@chudequebec.ca (M.-C.B.); marie-claude.brisson@chudequebec.ca (M.-C.B.); 3 Population Health and Optimal Health Practices Axis, CHU de Québec Research Center-Université Laval, Québec, QC G1S 4L8, Canada; amel.baghdadli@crchudequebec.ulaval.ca; 4 School of Psychology, Université Laval, Québec, QC G1V 0A6, Canada; gabriel.demers.7@ulaval.ca; 5 Division of Pediatric Hematology-Oncology, CHU de Québec-Université Laval, Québec, QC G1V 4G2, Canada; bruno.michon.med@ssss.gouv.qc.ca; 6 Reproduction, Mother and Youth Health Axis, CHU de Québec Research Center-Université Laval, Québec, QC G1V 4G2, Canada; 7 Faculty of Medicine, Université Laval, Québec, QC G1V 0A6, Canada; 8 Oncology Axis, CHU de Québec Research Center-Université Laval, Québec, QC G1V 4G2, Canada

**Keywords:** acute lymphoblastic leukemia, antineoplastic agents, cancer, child, medication adherence, qualitative research

## Abstract

Acute lymphoblastic leukemia (ALL) is the most common type of cancer in children. Treatment includes home-based oral chemotherapies (OCs) (e.g., 6-mercaptopurine and dexamethasone) taken for 2 to 3 years. The management of OC can be challenging for children and their parents. However, the multifaceted experience of families with children taking OC for ALL is largely undescribed. We report the experience with these OCs from the parents’ perspective. We conducted a qualitative descriptive study. Semi-structured interviews were conducted with the parents of children with ALL aged < 15 years, followed in a specialized university-affiliated center. The interviews were fully transcribed and thematically analyzed. Thirteen of the seventeen eligible parents (76.5%) participated in the study. The parents’ motivation to follow the recommendations provided by the multidisciplinary care team regarding OC was very high. The quantity and the quality of the information received were judged adequate, and the parents reported feeling knowledgeable enough to take charge of the OC at home. Adapting to the consequences of OC on family daily life was collectively identified as the biggest challenge. This includes developing and maintaining a strict daily routine, adapting to the child’s neurobehavioral changes during dexamethasone days and adapting family social life. Our findings have several implications for enhancing the support offered to families with home-based OC for ALL. Supportive interventions should consider the family as a whole and their needs should be regularly monitored. Specific attention should be paid to the development and maintenance of a routine, to the parental burden, and to the emotional impact, especially regarding dexamethasone.

## 1. Introduction

In North America, acute lymphoblastic leukemia (ALL) is the most frequent cancer among children < 14 years old [1]. The 5-year overall survival is approximately 90% [2,3]. This remarkable progress results from the intensification of chemotherapy and the refinement of risk-group stratification [2,3]. The regimens of treatment for children newly diagnosed with ALL typically take place in successive phases and include systemic chemotherapy (parenteral and oral) as well as central nervous system-directed therapy (intrathecal chemotherapy ± cranial radiotherapy) [3,4]. Corticosteroids (dexamethasone or prednisone) and 6-mercaptopurine (6-MP) are among the mainstay drugs used for preventing ALL relapse [3,4,5]. This requires that children take intermittent or daily doses of corticosteroids and 6-MP prescribed as oral chemotherapy (OC) at home for 2 to 3 years [3,4].

The use of these OCs is accompanied with recommendations to ensure their safety and efficacy. For instance, patients are commonly instructed to take 6-MP during the evening on an empty stomach (1 h before or 2 h after meals or snacks), without dairy products, and to handle it with gloves [6]. Corticosteroids, for their part, have been associated with psychological and physical side effects, including emotional lability, irritability, aggressivity, anxiety, depressed mood [7,8], weight gain, hyperglycemia, and hypertension [9]. Following these administration recommendations and coping with these side effects in the long term can be challenging for children and their parents. A study conducted among 441 children with ALL from diverse ethnicities reported that 43.8% of them did not take at least 95% of their prescribed 6-MP doses [10]. This study also supports the withdrawal of the 6-MP restrictive recommendations (i.e., taking it in the evening and without food or dairy products) as this did not significantly affect systemic exposure to the drug or increase the risk of relapse but did enhance adherence. The importance of high adherence to 6-MP in pediatric ALL was emphasized in a large study showing that missing ≥5% of the prescribed doses is associated with an increased risk of relapse (hazard ratio, 2.7; 95% confidence interval, 1.3–5.6) [5]. In a recent study, participants (49 parents and 15 patients with ALL) reported a median of four barriers to 6-MP adherence [11]. These were multifaceted, being associated with the parents’ and the patient’s capabilities, motivation, and environment. The most frequently reported barriers were to do with finding out what the medications were and meeting with other parents or patients. In a second analysis, these authors found that parents and patients with ALL were favorable to a mobile health intervention to support 6-MP adherence [12].

Some qualitative studies have provided insights into the journey of families with ALL children [13,14,15,16,17], but the specific experiences and needs regarding home-based OC are scarcely documented. One qualitative study focusing specifically on the lived experience with dexamethasone among children with ALL, assessed at two time points during the home-based therapy, indicates that the psychological effects of this treatment are profoundly disturbing for the child and the entire family [18,19]. A recent mixed-method study focused on parents’ challenges with OC administration, including 6-MP but excluding dexamethasone, during the maintenance phase [20]. The difficulties reported in administering the OC were related to their formulation and taste, side effects, required lifestyle adjustments, the child’s cooperation, and the parental burden associated with this new responsibility. A thorough description of the multifaceted experience of families specifically with both the 6-MP and the dexamethasone administered at home is lacking. This qualitative study aimed to describe the parents’ experiences with home-based 6-MP and dexamethasone prescribed to a child diagnosed with ALL.

## 2. Materials and Methods

### 2.1. Study Design and Conceptual Framework

We conducted a descriptive, qualitative study to gain an in-depth understanding of the experiences and needs regarding OC from the view of the parents [21]. The conceptual framework guiding this study was based on the ABC taxonomy of adherence and the Information-Motivation-Behavioral skills (IMB) model [22,23]. The ABC taxonomy depicts the components of medication adherence. When applied to home-based OC, these components include the initiation of OC, the implementation (respect for administration recommendations) and persistence for the prescribed duration (approximately 2 years) [22]. The IMB model conceptualizes factors that could influence the adoption of health-related behaviors such as medication-taking. This model postulates that an individual must know why and how to take the medication *(Information)*, become motivated to take the medication *(Motivation)*, and be capable of overcoming barriers and developing strategies (*Behavioral Skills*) [23].

### 2.2. Study Setting and Population

This study was conducted at the CHU de Québec-Université Laval, a network of teaching hospitals offering health services to the eastern population of Québec (Canada), including specialized and ultra-specialized pediatric care, where about 20 children are annually diagnosed with de novo ALL.

The study was conducted among parents of children <15 years old receiving a first-line pediatric ALL treatment protocol of the Dana-Farber Cancer Institute (ClinicalTrial.gov identifiers: NCT03020030), which globally consists of the following main successive phases: induction, consolidation I, central nervous system therapy, consolidation II, and continuation. Recruitment was performed between June 2019 and August 2019 at the outpatient clinic. To be eligible, parents had to: (1) be directly involved in managing the OC of a child undergoing consolidation or continuation phases (3-week cycles—6-MP taken on days 1 to 14 and dexamethasone taken on days 1 to 5—for approximately 2 years [3]) and (2) be able to participate in an interview in French. A member of the medical team presented this study as a study on the parents’ experience of OC at home to all eligible parents during a follow-up visit. The research team then explained the study in person or over the telephone and solicited the participation of parents who agreed to be contacted for the study. This study was approved by CHU de Québec-Université Laval Ethics Board (2020-4698). All the participants signed a consent form and received CAD 20 in compensation.

Patients and their caregivers at our institution are provided with education and support from a multidisciplinary team. Oncologists, pharmacists, and nurses are involved in the family’s initial teaching regarding OC. This includes the treatment plan, its aim, and specific information on each OC (e.g., dosing, side effects, and tricks to administer OC). The specific recommendations on OC and the general precautions with chemotherapy are also presented (at time of the study, taking 6-MP during the evening on an empty stomach without dairy products) and limiting potential sources of infection during periods of neutropenia (e.g., avoiding visiting a person with a contagious infection, avoiding large group meetings, and attending schools). Families are provided with written information (e.g., a standardized information sheet on OC, a daily dosing calendar, and a handout summarizing recommendations for the patient). During home-based OC phases, most patients have weekly appointments with the oncologists at the pediatric oncology clinic. They meet with the pharmacists at each of the OC refills, provided free of charge. As required, patients and their caregivers can also contact the nurse navigator and get services from other providers, including social workers, psychologists, nutritionists, physiotherapists, specialized educators, etc.

### 2.3. Data Collection

We conducted individual semi-structured interviews. The data collection and analysis were performed by a multidisciplinary team interested in pediatric oncology and the optimization of oral anticancer treatments. The interview guide was structured according to our conceptual framework (Appendix A). The principal investigators (I.L. and S.L.) were present during the first two interviews to ensure the interview guide’s accuracy. As only minor adjustments were made to the guide, these interviews were included in the analysis. The interviews took place at the hospital or by telephone and were audio-recorded and fully transcribed. The data collection was performed by five members of the research team (E.C.-B. and E.N., M.Sc. pharmacy candidates; G.D., psychology student; S.L., Ph.D. trained in social and cultural anthropology and epidemiology; and I.L., Ph.D. and part-time pharmacist at the in-patient pediatric oncology unit). None of the interviewers knew the participants before the study.

### 2.4. Analyses

The transcripts were thematically analyzed using a codebook developed through a validation process inspired by continuous thematic analysis [24]. Two research team members (E.C.-B. and E.N.) independently coded one interview using a first version of the codebook. This initial version of the codebook was developed based on the conceptual framework, the interview guide, the notes taken during the interviews, and also what emerged from the data. Based on this first coding experience, a second version of the codebook was produced and used to independently code four interviews by four research team members assembled in pairs (E.C.-B.–G.D., E.N.–G.D., and G.D.–A.B., M.Sc. trained in pediatric medicine and epidemiology). The coding of each pair was compared. Discrepancies were minor, and adjustments were made to the codebook. Using the final codebook, the remaining interviews were coded by two research team members (G.D. and A.B.) using QSR International Pty Ltd. (2018) NVivo Pro (Version 12). Based on the summaries of each code, the team identified emerging themes and their relationships. A professional translator translated the participants’ quotations into English. The participants were not solicited to provide feedback on the findings. As a validation process, to assess the credibility and transferability of our findings, these were shared and discussed with four parents who did not participate in this qualitative study. These parents are involved in managing OC for a child with ALL and are members of an advisory committee guiding our research team in studies of OC in ALL. The parents on this advisory committee met the same eligibility criteria as those in this qualitative study (see Section 2.2. Study Setting and Population). The advisory committee was presented with the main results of our analysis during a two-hour meeting, and the parents were invited to freely comment on the accuracy of these results based on their own experiences.

## 3. Results

**Figure 1:**
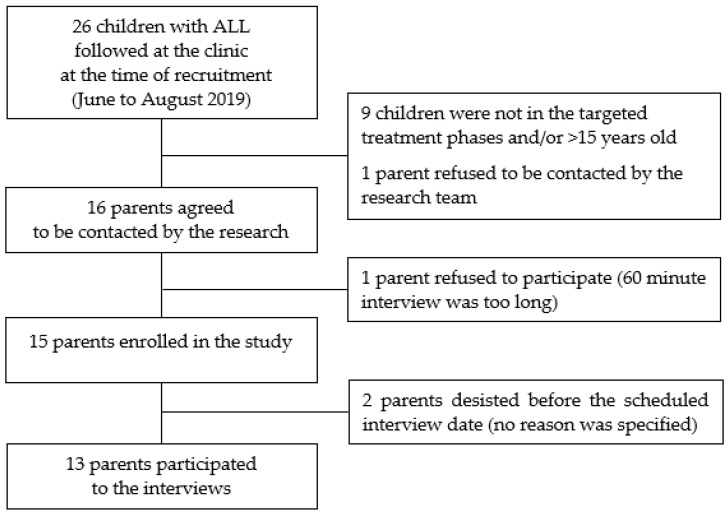
Study participants.

Seventeen of the twenty-six children with ALL followed at the outpatient pediatric oncology clinic at the time of the recruitment were <15 years old and in the treatment phases targeted for the study. Among the 17 eligible parents, 13 completed the interview (participation = 76.5%) (Figure 1).

The majority of the parents who participated in the study were women (11/13), and the average age was 38.2 years (25–52) (Table 1). The interviews lasted on average 50 min (range: 34–60 min). We identified three main themes of the parents’ experience: parents’ motivation toward adhering to home-based OC; parents’ knowledge; and the consequences of OC on family daily life.

### 3.1. Parents’ Motivation toward Adhering to Home-Based Oral Chemotherapy

Some parents first viewed returning home with OC as a stressful time in the treatment trajectory (Table 2, quotation#1). Nevertheless, all the parents ultimately felt relieved to be at home. From that point on, they felt responsible for their child’s healing (quotation#2); their goal was to reach the end of the treatment and follow the instructions carefully. This motivation was reflected in high self-reported adherence: all the participants had initiated the treatment and intended to persist for the prescribed duration. When a dose was occasionally missed or given with a delay, it was reported as unintentional. Few parents were able to list administration precautions (e.g., wearing gloves or not taking with dairy products) spontaneously or describe how these were applied, indicating that adherence to these instructions may be less optimal. However, this was not related to their motivation level.

### 3.2. Parents’ Knowledge about Oral Chemotherapy

The parents reported that the information about OC was verbally transmitted by pharmacists, and sometimes by nurses or physicians, on initiation and during follow-ups. They were also provided with written information sheets and calendars. Although reported as precise and helpful, parents felt lost, overwhelmed, and unable to retain all the information and instructions when first briefed about OC (Table 3, quotation#3). Follow-ups were an opportunity to meet with healthcare professionals, especially the pharmacist, and obtain advice for the next cycle or to be reminded of important information (quotation#4). Although the information provided during follow-ups was perceived redundant, the parents considered this essential to strengthen their knowledge, reassure them, and help them remember questions they had at home. Many parents mentioned that receiving both oral and written information was essential for retention. The majority said that the general information provided by the medical team was adequate in terms of quantity and quality. The parents were able to report the general goal of OC, and some were able to explain the drug mechanisms of action in their own words. However, the parents’ knowledge of OC seemed to be mainly centered on its management in their everyday life.

#### 3.2.1. Dexamethasone

The goal of dexamethasone was often unclear for the parents; some considered it as chemotherapy, others as a supportive medication (quotation#5). However, the parents considered dexamethasone as important as 6-MP. The parents reported respecting the recommendation to give dexamethasone with food, although the reasons remained unclear for some. Most parents extensively discussed the side effects of dexamethasone. The emotional and mood changes, including sadness, irritability, anxiety, and the child’s need for isolation, were the most disturbing ones. Even though they were usually informed of these potential side effects at the start of treatment or experienced them during hospitalization, the parents did not expect their intensity; instead, they became aware of these reactions as their child experienced them.

#### 3.2.2. 6-Mercaptopurine

The 6-MP was associated with administration complexity (quotation#6). While the parents were generally aware that 6-MP should be taken under fasting conditions and during the evening, the recommendations regarding its administration without dairy products and handling precautions were unclear for some.

### 3.3. Consequences of Oral Chemotherapy on Family Daily Life

Adapting to the consequences of OC on family daily life was reported by the parents as being the biggest challenge. The treatment enforced the developing and maintaining of a new routine, required adapting to the changing child’s behavior, and affected their social life.

#### 3.3.1. Developing and Maintaining a Routine

All the parents talked about the need to develop a new routine to integrate the OC into family daily life. According to the parents, the routine was an adaptation of their everyday life, including administering drugs at specific hours, planning meals and bedtime, preparing doses in advance, managing the treatment calendar, and dealing with the side effects.

The parents reported that developing an effective routine takes around two to four cycles (approximately 6–12 weeks). They talked about the importance of being assiduous at the beginning of the treatment before the routine was established (Table 4, quotation#7). The parents found this adaptation period difficult and restrictive (quotation#8). Most felt that it was important that one parent take the lead in the treatment management (quotation#9), even when the parents were living apart (quotation#10). Advice from the oncology team also helped in some cases to develop a routine adapted to the families’ lifestyles. Once developed, the routine was perceived as essential for the entire 2 years to ensure the smooth running of the treatment. A routine allowed remembering critical OC administration elements and diminished the stress related to potential forgetfulness and mental load. The parents talked about the difficulties of resuming the OC at the beginning of each new cycle due to the loss of routine during the weeks of treatment break, and the difficulty in maintaining a routine during family outings or vacations (quotation#11). Respecting the daily routine allowed some families to involve the children in their treatment more significantly. Overall, establishing a routine was perceived as both a helpful strategy and a source of frustration. The parents mainly presented the routine as a facilitator for medication management (quotation#12). However, the implications of a routine on everyday family life were also described as constraining (quotation#13).

#### 3.3.2. Adapting to the Child’s Behavior

Globally, the children understood that they were sick and needed treatment. Various factors, such as age or personality, seemed to affect their perception and acceptance of the ALL treatment. The majority of the children had a role to play in drug administration and were described by the parents as being helpful and diligent. Implicating the children in their treatment was one of the strategies used by parents to facilitate medication administration and to help them cope with their condition. Being responsible for their therapy seemed to empower the children, as they felt more in control (Table 5, quotation#14).

The changes in the children’s behavior resulting from taking dexamethasone were disturbing for the parents. Many reported that their children’s behavior changed rapidly when exposed to dexamethasone, which causes emotional lability and, in some cases, a depressed mood, effects that disappear when dexamethasone is stopped (quotation#15). Most children seemed to be aware of the mood changes induced by dexamethasone, and some were more affected by them. Some parents said they respected the children’s need to be alone during this time (quotation#16); others tried to maximize social interactions for their children (quotation#17). The parents mentioned that developing a proper understanding of their children’s condition and adapting their families’ dynamics was essential. Some parents avoided talking about sensitive subjects and were more permissive during the dexamethasone taking, mentioning that every family member needed to do his part. Explaining and discussing the child’s condition with their siblings helped their understanding of the situation (quotation#18). The support received from the oncology team for identifying and managing the dexamethasone side effects was essential for some parents.

#### 3.3.3. Adapting Relationships Outside the Family

Seclusion was prevalent among families due to their taking of all the steps necessary to ensure their child’s recovery. Preventing infections in their immunosuppressed child meant limiting outside contacts (Table 6, quotation#19). The parents secluded and often refrained themselves from leaving the house to take care of their children. This decision either arose from the parental choice to ensure proper OC administration or was dictated by the child’s demands or behaviour. Moreover, the emotional confinement of the parents was very noticeable (quotation#20). The children also experienced seclusion but in a different manner. Some children, feeling that their mood and reactions were changing during dexamethasone, preferred to isolate themselves to spare others their difficult behavior (quotation#21).

## 4. Discussion

To the best of our knowledge, this is the first qualitative study to specifically describe the subjective parental experience with the concomitant use of home-based 6-MP and dexamethasone, two cornerstone drugs for lasting recovery from ALL. Studying this combination enabled us to capture both the specific difficulties of each drug and their cumulative burden. The findings provide rich and detailed insights into the families’ experiences with home-based OC. The parents felt very responsible for managing the OC at home and were highly motivated to follow the recommendations. They also reported being generally well informed, having gained the required knowledge to take charge of the home-based treatments throughout the treatment course. However, the parents collectively reported that adapting to the consequences of the OC on family daily life was the biggest challenge. The treatment enforced the development and maintenance of a strict family routine, required adapting to changes in the child’s behavior during dexamethasone, and required adapting relationships, both inside and outside the family.

The parents’ motivation toward adhering to 6-MP and dexamethasone recommendations was very high; one parent said that managing the treatment at home correctly was his/her “mission”. This is in direct line with the findings issued from a qualitative study where the parents of 12 children with ALL described their overall journey as “Being there”, meaning “I’ll be there for you” and “Whatever happens, you can count on me, I will never let you down” [13]. Our findings indicate that the parental role, which may change in the context of a potentially fatal illness such as ALL [16,17], also accurately applies to the home-based phase of treatment, when the parents embrace new responsibilities regarding their child’s treatment. Even if highly motivated, entering this new phase was first perceived as a stressful step by some parents. A previous qualitative study also showed that the parents’ representations of the first transition from hospital to home with an ALL child were antagonistic, characterized by both apprehensions and relief [25]. Thus, this transition may be a critical time when parents need support to adapt to their new responsibilities, including OC management, and to a new family reality.

Despite the amount and the complexity of the information that needed to be assimilated, the parents integrated crucial information in order to self-manage home-based OC. They were generally very satisfied with the information received, as previously documented for the more general type of information received in the context of pediatric cancer [15,26]. It is likely that understanding the aim and general mechanisms of the action of 6-MP and dexamethasone contributed to enhancing the parents’ motivation towards initiating, implementing, and persisting with the OC. This may also reinforce the parents’ perception that OCs are just as important as intravenous ones. However, some parents did not clearly understand the purpose of dexamethasone and were unprepared to deal with the emotional and behavioral changes caused by this drug on their child. Specific attention should be paid to dexamethasone, and crucial information regarding its potential impact [18,19,27] should be provided well before the occurrence of side effects.

Being motivated and well-informed does not mean that this step in treatment was easy to live through for the families. Some studies have looked at the overall journey of families with a child diagnosed with cancer, including ALL [13,14,16,17]. However, our study brings a novel insight into the challenges faced by parents and their children which is specific to integrating the recommendations for the intake of OC at home. The parents’ testimonies reveal that translating the use of this motivation and knowledge into optimal treatment results from constant and daily efforts involving the entire family. The repercussions were felt within the family but go much further as the family’s place within the social network was also affected. Establishing a routine was one of the main strategies used by the parents to integrate the treatment and ensure it was taken appropriately. This strategy, which echoes research on the importance of habit for adherence [28], was adopted intuitively by the parents. However, developing an automatic habit may be challenging in the context of intermittent OC, especially if the administration precautions are complex. This new routine was also described as constraining, and some parents felt burned-out by following a strict routine. Parents fully embracing the responsibility of administrating OC have also been described in other studies [20]. Our findings highlight, however, that this responsibility is mainly endorsed by one of the parents, either because they were single parents or because they were afraid to delegate this responsibility, or it made it easier to keep track of the drug administration. The child’s need for seclusion and the changes to the family dynamics because of the routine also contributed to parental fatigue.

One of the biggest challenges encountered by the families was adjusting to the emotional and psychological changes associated with dexamethasone [18,19]. The parents, like some of the children, found it profoundly troublesome. Some of the parents modified their parenting style, and the family dynamics changed to accommodate mood changes and minimize their impact [19,27]. In addition, this led to seclusion, sometimes initiated by the children themselves to spare others from their difficult behavior during the weeks of dexamethasone. The physical and psychosocial effects of dexamethasone on families have already been studied [7,8,18,19], and our findings also stress this aspect of the treatment burden from the parents’ perspective. Our results go further by suggesting that, in the context of dexamethasone, social isolation is a family choice that still could cause distress. Again, greater attention needs to be paid to the global lived experience of dexamethasone for intervention development.

Our study has some limitations. Despite relatively high participation (13 of 17 eligible participants), our sample size was limited due to the number of children diagnosed each year with ALL at our hospital (~20 cases). However, the saturation of the main themes was reached as most of the participants shared the same issues. In addition, four parents involved in the management of the OC who are members of our research advisory committee validated that the study findings accurately reflected their own experience. This collective exercise, however, led parents from the advisory committee to emphasize that the preponderance of certain experiences and needs could vary according to the child’s health and age, family structure, and time in the treatment trajectories. The potential impact of these differences on OC experience could not fully be assessed in this study. Our results are more likely to apply to families followed in a specialized hospital with access to a multidisciplinary oncology team. Nevertheless, different pediatric protocols are commonly used to treat ALL, which may differ in 6-MP administration precautions, the use of 6-MP intermittently versus daily therapy, and the frequency of follow-up visits [3,4]. This could affect the generalizability of some of our findings, such as the ease of integrating OC in existing routines and the opportunities to obtain advice. Similarly, the education and recommendations provided with OC can also vary between health centers [10,29].

### Implication for Support Interventions

The needs regarding OC are multifaceted and could be experienced by all family members. We identified three main aspects of the parents’ experiences having direct implications for intervention development. First, taking on the responsibility of self-managing these treatments at home requires integrating complex information. The parents’ testimonies reveal that educational interventions should be delivered gradually using various modes and tools. Offering frequent opportunities to address questions and difficulties as they evolve throughout the treatment trajectory is also crucial. Second, integrating these complex treatments and precautions for two years was very demanding for the whole family. Developing and maintaining a routine was perceived as an essential and effective strategy for managing medication that was also burdensome. The medical team should make accommodations, when possible, in medication intake to ease the integration of the OC into the family’s lifestyle and lessen the burden. Finally, the impact of the treatment goes far beyond drug management, and parents reported some psychosocial needs that were sometimes only partially met for some of them. The neurobehavioural effects of dexamethasone, concerns about parental attitudes to adapting to the child’s changing behavior, parental onus and fatigue, and the social isolation of the child and family were reported as deeply troubling. These difficulties should be monitored and addressed proactively by involving the multidisciplinary team.

## 5. Conclusions

The experience and needs regarding OC are multifaceted; it is, therefore, essential that support interventions be as well. Our team is currently developing a program consistent with this vision in close collaboration with parents and healthcare and psychosocial professionals. With the emergence of oral-targeted therapies for pediatric cancers, such treatment-related interventions to support families should be prioritized.

## Figures and Tables

**Table 1:** Parents’ and children’s characteristics.

Characteristics of Parents (*n* = 13)	
Age (years), mean (min-max) ^1^	38.2 (25–52)
Female (n)	11
Distance from home to the treatment center (km)	
<50	10
≥50	3
Highest education level (n)	
High School	2
CEGEP	6
University	5
Work situation (n)	
Paid work	6
Work interruption	6
Unemployed	1
Living in a couple (n)	11
Parents having at least another child (*n*)	9
**Characteristics of children diagnosed with ALL (*n* = 13)**	
Age (years), mean (min-max)	7.5 (3–13)
Female (n)	7

^1^ Age missing for one parent; CEGEP: Professional and general collegial education. Collegial level represents 2–3 years after high school but before university-level education.

**Table 2:** Exemplar quotations from interviewed parents for the theme: Parents’ motivation toward adhering to home-based oral chemotherapy.

Quotation 1	“[…] I knew that I could handle it. Like I said, it was a bit more stressful…at first, you want to make sure you’re doing it properly…that you don’t forget anything, but…I really felt like it was my mission…I mean, it was my priority.” (NID11)
Quotation 2	“In terms of…the responsibility, I mean obviously at the hospital the nurses…administer them (*the medication*) and they remember to do it according to a set schedule, here we have to…take on that responsibility for ourselves, but it’s doable.” (NID 10)

**Table 3:** Exemplar quotations from interviewed parents for the theme: Parents’ knowledge about oral chemotherapy.

Quotation 3	“[…] everything was perfect, it’s just that…when you leave the hospital your brain is crammed with all kinds of things that you mustn’t forget […] since there’s a lot of information, you can’t retain it all, so yes, everything was well done, everything was relevant, everything was in the right place…even if you put more *(information)* before or more after, I don’t think it would change anything, I mean there’s just so much to think about and remember, but I do think it was all at the right time.” (NID06)
Quotation 4	“...I like that the pharmacist comes to see you with the medication and really explains…the steps and everything that needs to be taken. And then…if you have questions, I often have questions and I feel like I get a lot of support to…make sure I’m administering the medication properly. […] I just like it because, I haven’t necessarily forgotten, but just getting the reminder to make sure that […] everything I’m doing at home…I’m doing properly. Because I don’t want to, you know, it’s a big responsibility, and I want to make sure I’m doing it properly […] I just like getting a little ‘refresher’ each…time.” (NID09)
Quotation 5	“…What I understand about these two drugs is that it’s very important to take them because it’s part of…the chemotherapy, especially the Purinethol [i.e., 6-MP], which lowers her blood count, which is what you want. […] For the Decadron [i.e., dexamethasone], I think it’s for protection […].” (NID06)
Quotation 6	“Because with Purinethol [i.e., 6-MP] it’s more complicated, you can’t eat two hours before you take it and for an hour after you take it…We had to work it into the family routine. We talked to the pharmacists about it, because they always told us to take it at night. At first the doctors told us to take it at night. We talked to the pharmacists and came to an agreement that we could give it to him when he got home from school, so he wouldn’t eat two hours before or one hour after, so before dinner. That was easier to manage, with snacks in the evening or whatnot. Otherwise sometimes we’d have to wake him up to give him his medication and…I would say that the two weeks of Purinethol [i.e., 6-MP], those are the ones that when we finish the two weeks, we’re like ‘Ahhhh’. It’s like our week off.” (NID04)

**Table 4:** Exemplar quotations from interviewed parents for the theme: Developing and maintaining a routine.

Quotation 7	“…It’s more in terms of the daily routine that it changes a bit, well a lot, actually, but I mean, the inconvenience that we see is that you have to really be on it. When I told you that we don’t have a routine at home, to really have that kind of a routine, we really had to work hard every day.” (NID02)
Quotation 8	“…It’s definitely a bit more stressful, because it means that it’s not just the nurses and doctors who are in charge of the treatment, it’s us. So I want to make sure I’m not forgetting anything, that I’m doing it properly, that I’m doing it at the right time…It definitely is an added stress in my life, and it’s already a stressful time. So it’s definitely stressful, I can’t say that it hasn’t made my life more stressful. Yes, it’s made my life more stressful. […] I know it will be that way, it will be a part of my life until 2021 (laughs)…But I’m hopeful that with time it will be much more routine, that it will just, you know, be a part of, so that it takes up less mental space, it’s still pretty new…” (NID09)
Quotation 9	“…It’s pretty much on my shoulders, this one (*the treatment),* but at the same time it’s okay because maybe it’s easier if there’s just one person managing it […] You know maybe it’s for that reason, so that you don’t think ‘Oh, maybe they’ll (*referring to the other parent)* do it.’ Maybe it’s easier and maybe there are less mistakes if there’s one person assigned to the task, instead of two people thinking they’ll do it together and then neither of them does it […].” (NID12)
Quotation 10	“[…] Henri doesn’t have both of his parents together, so we had to figure out a system to make sure we didn’t make any mistakes, since I’m the one who handles the medication, so that’s my job, and then when he’s at his dad’s place, I get it all ready for him, but…I’m, as I was saying, schedules… I wasn’t particularly worried about it. […] With the Decadron [i.e., dexamethasone] and the Purinethol [i.e., 6-MP], obviously sometimes he’s at his dad’s, sometimes he’s here…I make him a schedule in advance for when he’s at his dad’s, and anyway he’s never there for more than 3 days, so I make him a daily schedule, and I put what he needs and I prepare his doses so he just has to follow…the schedule that I set for him, but I’m the one in charge of the schedule, so that way we don’t make any mistakes.” (NID10)
Quotation 11	“[…] Sometimes, when we’re not at home, say, and then our routine is a little different and we have the chemo with us, sometimes it’s maybe less of a reflex, so I put alarms on my phone, just in case […].” (NID09)
Quotation 12	“…we just have to remember, but we get into the habit pretty quickly, it becomes a reflex, we give it to him, we administer it, and then…we don’t think about it anymore.” (NID04)
Quotation 13	“…it was to try to line up the sleeping, to try to have a semblance of sleep, in between the gavage feeding and the Purinethol [i.e., 6-MP] and so on…It wasn’t fun. Now it’s not as bad, but yeah, you have to wait, sometimes I’d like to go to bed at 8 pm, fall asleep at the same time as the little guy, but I know that at 10 pm, for example, I have to give him his Purinethol [i.e., 6-MP]… it’s definitely a downside, to have to put off bedtime to give him his medication.” (NID 13)

All names are fictional. They were chosen to make the excerpts easier to read and to ensure participants remain anonymous.

**Table 5:** Exemplar quotations from interviewed parents for the theme: Adapting to the child’s behavior.

Quotation 14	“…I feel like it gives her a chance to look after herself, like she’s in charge of her healing, not us. It’s part of her daily routine, and yes it’s the whole family’s daily routine, but it’s especially her daily routine. To be fully in control for taking her medication, we’re always after her, you know, ‘Have you taken your meds?’ ‘Yes mom’ (imitating her daughter), but it’s funny, but she sets reminders, she has a little cellphone, so she sets reminders for taking her medication, but yeah, I think it’s really in terms of it being her responsibility, of feeling that she’s in control.” (NID02)
Quotation 15	“[…] Decadron [i.e., dexamethasone], the bad mood, irritable, full of sadness, so when it’s Decadron [i.e., dexamethasone] week… we adapt, you know… she cries more and she’s sad, and then it’s over, ‘Ah, she’s back to her usual self.” (NID05)
Quotation 16	“[…] I always ask Raphaelle how are you feeling, how are you doing, and sometimes we know she doesn’t feel like talking and we let her be, you know. Or sometimes she just stays in her room, so we just need to leave her…let her be in her bubble. I make sure that she’s doing okay, that she’s feeling okay, but you don’t want to insist to much, you need to give her some space, to process things, you know sometimes she wants to be alone and it’s not because she’s not doing well, she just needs space, she needs to be in her world, and it’s important to respect that..” (NID11)
Quotation 17	“…And at the same time we respect that because she knows how she is, so she doesn’t want to impose her moods on other people, and I know that’s often why she goes to her room, but you know, we still go and get her. Or when she has friends over too we try to have friends over to take her mind off things. But at the same time, she says, mom I look so grumpy, I don’t feel like seeing my friends. We understand, we’d feel the same way, so we don’t insist, but we really try to do things together to take her mind off it.” (NID02)
Quotation 18	“It puts a damper on the mood at home, for sure. We have to explain it to his big brother, that it’s not Gabriel’s fault, that it’s because of the medication, it’ll end. So maybe some minor conflicts like that because of the Decadron [i.e., dexamethasone], the side effects of the Decadron [i.e., dexamethasone].” (NID07)

All names are fictional. They were chosen to make the excerpts easier to read and to ensure participants remain anonymous.

**Table 6:** Exemplar quotations from interviewed parents for the theme: Adapting relationships outside the family.

Quotation 19	“…When we know that her blood is low, we stay away from people who are sick, we don’t […] go to all-you-can-eat buffets so that she doesn’t catch any bacteria, we’re careful about where we go, we don’t go too far, so that if something happens, we’re close to a hospital, if she isn’t feeling well she tells us and we change our plans accordingly, you know it’s changed our lives a bit, so we do what we have to do to make sure she can fully heal.” (NID11)
Quotation 20	“…When you’re going through that, besides talking to your partner, you’re going through it alone. You know, your best friend probably doesn’t have any experience with leukemia, so you don’t really have any friends you can talk to, besides your partner.” (NID05)
Quotation 21	“…She definitely has a tendency to go off on her own because she knows she can be grumpy, so we really try to get her out with us [...]. Because otherwise, she locks herself in her room with her headphones, I mean she makes bracelets or whatever, she’s still doing things, but I mean she has a tendency to isolate herself.” (NID02)

## Data Availability

The data that support the findings of this study are available from the corresponding authors upon reasonable request.

## Appendix A

Interview guide

1. Introduction
2. The child and his(her) treatment
  - How old is your child?
  - At what age was your child diagnosed with acute lymphoblastic leukemia?
  - What is your child’s current treatment?
3. Information
  - What have you been told about your child’s current stage of treatment at home?
  - How was this information provided to you?
  - What did you think of the way this information was presented to you?
  - Did you consult any other sources of information? If so, which ones?
  - What was the most helpful to you?
  - How sufficiently informed do you feel to manage your child’s treatment at home? Why or why not?
4. Motivation
  - At the very beginning of the treatment, how did you view this new stage of treatment happening at home?
  - What is your perspective on this stage now?
  - How do you envision the continuation of this treatment until the end?
  - What are the benefits to you of following the medical team’s recommendations regarding this treatment at home?
  - What are the disadvantages to you of following the medical team’s recommendations for this treatment at home?
  - What would motivate you to pursue the treatment until the end? What could, on the contrary, affect your motivation?
5. Behaviour
  - What was it like for you and your child at the very beginning of taking 6-mercaptopurine and dexamethasone at home? Was there a difference between the two medications?
  - How is it going now? Is there a difference between the two medications?
6. Capacity
  - At the very beginning, how did you feel about your ability to give this new treatment at home?
  - At first, what helped you to give this treatment at home?
  - At first, what made it more difficult to give this treatment at home?
  - Was it more difficult for any of the medications? Why?
  - You told me that at first [REPRHASE THE DIFFICULTY EXPRESSED]. Is it currently the same?
  - If you try to look ahead, between now and the end of the treatment.
    o What would make it easier to take the treatment?
    o What would make it harder to take the treatment?
  - How supported do you feel in managing this treatment at home?
7. General intervention
  - Overall, what has been most helpful for you to use 6-mercaptopurine and dexamethasone at home?
  - What should be done to help families like you to manage these medications at home?
  - What concrete form should this [REPEAT PARENT’S WORDS] take?
8. Conclusion
  - Is there anything else we haven’t talked about regarding the treatment at home and that you would like to discuss?
9. Acknowledgements
